# Prognostic significance of serum iron panel in breast carcinoma: correlation with histopathological grading and molecular immunophenotypes

**DOI:** 10.1007/s12672-026-04923-3

**Published:** 2026-03-27

**Authors:** Ashish P. Anjankar, Archana Dhok, Mohd Basheeruddin, Sana Qausain, Samarth Shukla, Namrata Choudhary, Rushikesh Pramod Ingole

**Affiliations:** 1 Department of Biochemistry, Jawaharlal Nehru Medical College, Datta Meghe Institute of Higher Education & Research (Deemed to be University), Wardha, India; 2 Department of Pathology,Jawaharlal Nehru Medical College, Datta Meghe Institute of Higher Education & Research (Deemed to be University), Wardha, India; 3 School of Allied Health Sciences, Datta Meghe Institute of Higher Education & Research (Deemed to be University), Wardha, India

**Keywords:** Breast carcinoma, Serum iron panel, Ferritin, Transferrin saturation, Histopathological grading, Molecular immunophenotypes, Prognosis

## Abstract

**Background:**

Breast carcinoma is the most common malignancy in women globally, with prognosis significantly impacted by histopathological grading and molecular immunophenotypes. Iron metabolism dysregulation plays a crucial role in cancer progression, and serum iron panel components have emerged as potential prognostic biomarkers, aiding in disease assessment and management.

**Objective:**

This review aims to explore the prognostic significance of the serum iron panel in breast carcinoma and its correlation with histopathological grading and molecular immunophenotypes.

**Methods:**

A thorough literature review was performed using peer-reviewed articles from PubMed, Scopus, and Web of Science. Studies examining the relationship between serum iron parameters (serum iron, ferritin, transferrin saturation, and total iron-binding capacity) and breast carcinoma subtypes, histopathological grading, and molecular immunophenotypes were analyzed for inclusion.

**Results:**

Serum iron parameters are altered in breast carcinoma but the results are heterogeneous. High ferritin levels are indicative of higher tumor grade, as well as increased tumor burden and poor outcomes. Transferrin saturation and TIBC exhibit different patterns. More aggressive subtypes, most notably triple-negative breast cancer, show greater iron dysregulation, suggesting a connection between iron metabolism and tumor characteristics despite inconsistencies in findings.

**Conclusion:**

Serum iron biomarkers, such as ferritin, could indicate cancer aggressiveness and subtype in breast cancer. However, the evidence is inconsistent and mostly observational. These markers may be viewed as adjuncts rather than definitive prognostic tools and would benefit clinically best from validation via large-scale prospective studies.

## **Introduction**

### Background

Breast carcinoma is a highly heterogeneous malignancy with distinct histopathological and molecular characteristics. It is the most common cancer among women worldwide and a leading cause of cancer-related mortality. The rising incidence of breast carcinoma is linked to genetic predisposition, hormonal influences, lifestyle modifications, and environmental factors [1]. Despite significant advancements in early detection and treatment, breast carcinoma remains a global health concern, necessitating continuous research to identify novel prognostic markers and therapeutic strategies. Several prognostic factors influence breast cancer outcomes, including tumor histopathological grading, molecular subtypes, receptor status, lymph node involvement, and systemic metabolic alterations. Among these, iron metabolism has gained attention due to its role in tumorigenesis, progression, and metastasis [2].

Iron is an essential micronutrient involved in oxygen transport, mitochondrial function, and DNA synthesis. However, dysregulated iron homeostasis can promote oxidative stress, DNA damage, and uncontrolled cellular proliferation, contributing to cancer development. Studies have revealed alterations in iron metabolism in breast carcinoma, suggesting its potential significance in disease progression and prognosis [3]. The serum iron panel, consisting of serum iron, ferritin, transferrin saturation, and total iron-binding capacity (TIBC), has been investigated for its prognostic relevance in breast carcinoma. These parameters provide insight into tumor biology, iron homeostasis, and disease outcomes. Evaluating the relationship between iron metabolism and breast carcinoma may enhance prognostic assessment and therapeutic decision-making, ultimately improving patient management [4].

### Objective and purpose of review

This review aims to explore the significance of iron metabolism in breast carcinoma and its potential role as a prognostic biomarker. Breast carcinoma is a heterogeneous malignancy influenced by various molecular and metabolic alterations, including iron homeostasis dysregulation. Iron is essential for numerous cellular processes such as oxygen transport, mitochondrial function, and DNA synthesis. However, its dysregulation contributes to tumor progression, metastasis, and therapy resistance through mechanisms involving oxidative stress, DNA damage, and uncontrolled cellular proliferation. Understanding the impact of iron metabolism in breast carcinoma is crucial for identifying novel prognostic markers and therapeutic targets [5].

A key objective of this review is to assess the role of iron metabolism in tumor progression and metastasis, as cancer cells often exhibit altered iron homeostasis that enhances their proliferation and survival. Additionally, this review examines the correlation between serum iron panel components-including serum iron, ferritin, transferrin saturation, and TIBC-with histopathological grading of breast carcinoma [6]. These iron-related biomarkers may provide valuable insights into tumor aggressiveness and disease prognosis. Furthermore, this review evaluates the association between iron metabolism and different molecular subtypes of breast carcinoma, such as luminal a, luminal B, HER2-enriched, and triple-negative breast cancer (TNBC), each of which exhibits distinct metabolic and iron regulation patterns that may influence treatment responses and clinical outcomes [7].

The purpose of this review is to provide a comprehensive analysis of iron metabolism and its prognostic relevance in breast carcinoma. By reviewing existing scientific literature, this study aims to enhance the understanding of how iron metabolism influences breast cancer progression and to identify potential biomarkers within the serum iron panel that could aid in disease stratification and prognosis [5]. Additionally, this review seeks to encourage further research into integrating iron metabolism-related biomarkers into clinical oncology for improved patient management. Finally, emerging therapeutic strategies targeting iron dysregulation, including iron chelation therapy and ferroptosis-inducing drugs, will be explored as potential interventions for breast cancer treatment [8].

## Role of iron metabolism in cancer progression

**Fig. 1:**
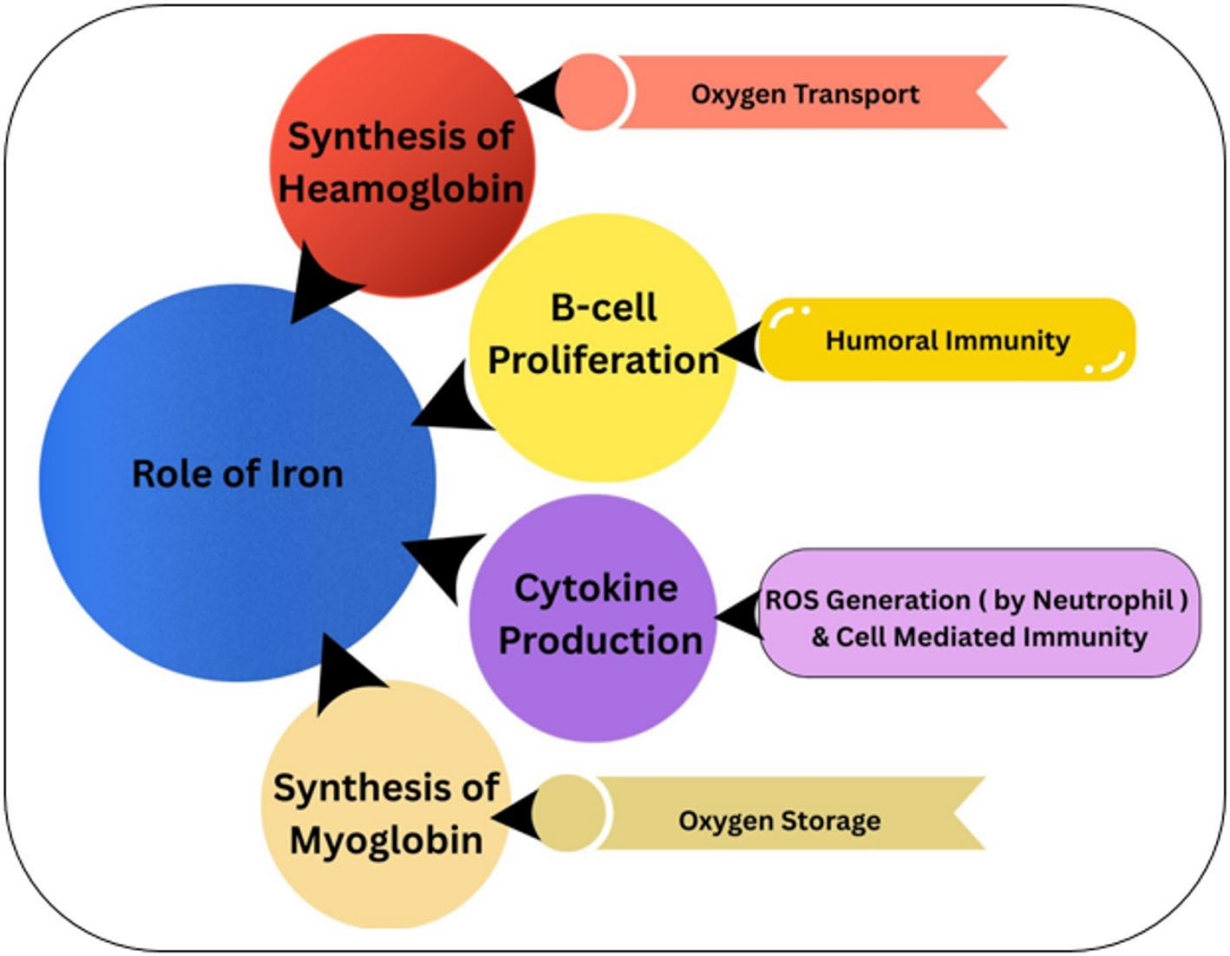
Iron Metabolism in Cancer Cells

Iron is an essential trace element involved in various cellular functions, including oxygen transport, energy production, and DNA replication see in Fig. 1. It plays a crucial role in cellular respiration and serves as a cofactor for numerous enzymes necessary for cell survival. However, dysregulation of iron homeostasis has been implicated in carcinogenesis, with cancer cells exhibiting an increased demand for iron to sustain rapid proliferation and metabolic activity. This phenomenon, often referred to as “iron addiction,” is characterized by heightened iron uptake and decreased iron efflux, leading to iron accumulation within tumor cells [9].

### Oxidative stress and DNA damage

One of the primary mechanisms linking iron metabolism to cancer progression is its role in oxidative stress. Iron participates in Fenton reactions, generating ROS such as hydroxyl radicals. These highly reactive molecules induce oxidative stress, causing DNA damage, lipid peroxidation, and genomic instability. Such alterations can trigger mutations in oncogenes and tumor suppressor genes, facilitating cancer initiation and progression. Excessive ROS production also promotes epithelial-to-mesenchymal transition (EMT), a process associated with tumor invasion and metastasis [10].

### Iron and angiogenesis in tumor growth

Iron plays a significant role in angiogenesis, a critical process for tumor growth and metastasis. It promotes the expression of vascular endothelial growth factor (VEGF), a key regulator of new blood vessel formation. Increased VEGF levels facilitate the supply of oxygen and nutrients to tumors, enhancing their expansion and metastatic potential. The iron-rich tumor microenvironment supports this pro-angiogenic signaling, further contributing to aggressive tumor behavior [11].

### Dysregulation of iron regulatory proteins

Iron homeostasis is tightly regulated by proteins such as transferrin, transferrin receptor (TfR), ferroportin, hepcidin, and ferritin. In breast carcinoma, altered expression of these proteins has been observed. Elevated transferrin receptor levels enhance iron uptake in tumor cells, while reduced ferroportin expression leads to iron retention. These alterations create an iron-enriched tumor microenvironment that fuels cancer progression. Understanding these mechanisms may provide novel insights into therapeutic strategies targeting iron metabolism in breast cancer treatment [12].

## Serum iron panel and its components

The serum iron panel consists of key iron-related parameters that provide insights into systemic iron metabolism and availability. Each component plays a crucial role in maintaining iron homeostasis and has been studied for its potential association with breast carcinoma see in Table 1. Serum iron refers to the amount of circulating iron bound to transferrin, the primary iron-transporting protein. Its levels fluctuate based on dietary intake, iron stores, and physiological demand [13]. In malignancies, including breast carcinoma, altered serum iron levels have been reported, with studies indicating both increased and decreased serum iron concentrations. Elevated serum iron may enhance oxidative stress and DNA damage, promoting tumor progression. Conversely, lower serum iron levels may reflect sequestration by tumor cells, increasing their iron availability for proliferation. Ferritin is the primary intracellular iron storage protein, regulating iron homeostasis by sequestering excess iron and preventing toxicity. Elevated serum ferritin levels are frequently observed in cancer patients, including those with breast carcinoma. High ferritin concentrations may indicate increased iron demand by rapidly proliferating tumor cells or reflect an inflammatory response linked to malignancy. Ferritin also plays a role in immune regulation and oxidative stress modulation, further influencing cancer progression [7].

Transferrin saturation represents the proportion of transferrin molecules bound to iron, serving as an indicator of iron bioavailability. Reduced transferrin saturation has been associated with aggressive tumor phenotypes, potentially due to altered iron transport mechanisms in cancer cells. Low transferrin saturation may reflect an imbalance between iron demand and supply, leading to iron sequestration in tumor tissues and contributing to an iron-enriched tumor microenvironment [7]. TIBC measures the capacity of transferrin to bind and transport iron. It is inversely related to serum iron levels and reflects iron mobilization efficiency. In breast carcinoma, decreased TIBC levels have been reported, suggesting iron overload or impaired iron mobilization within the tumor microenvironment. Reduced TIBC may indicate excessive iron retention in tumor cells, promoting oxidative stress, angiogenesis, and enhanced tumor aggressiveness. Understanding the role of these serum iron panel components in breast carcinoma could help refine prognostic assessments and therapeutic strategies. Identifying iron metabolism dysregulation patterns may lead to the development of novel biomarkers and targeted interventions aimed at modulating iron homeostasis in cancer treatment [4].

**Table 1:** Summary of Serum Iron Panel Alterations in Breast Carcinoma Subtypes

Component	Normal Range	Changes in Breast Cancer	Prognostic Implication	References
Serum Iron	50–170 µg/dL	Increased/Decreased (Variable)	Reflects tumor iron utilization	[14]
Ferritin	15–200 ng/mL	Elevated	Correlates with tumor burden and inflammation	[15]
Transferrin Saturation	20–50%	Decreased in aggressive tumors	Indicator of iron availability	[16]
TIBC	250–450 µg/dL	Decreased in cancer	Reflects iron overload status	[4]

## Correlation of serum iron panel with histopathological grading

Histopathological grading of breast carcinoma is a critical determinant of tumor aggressiveness and prognosis, based on factors such as tumor differentiation, mitotic index, and nuclear pleomorphism. Emerging evidence suggests that iron metabolism plays a crucial role in tumor progression, with variations in serum iron panel components correlating with different histopathological grades [17] is depicted in Table 2.

**Table 2:** Iron Dysregulation across Histopathological Grades of Breast Carcinoma

Histopathological Grade	Serum Iron	Ferritin	Transferrin Saturation	TIBC	Reference
Grade I	Normal	Slightly Increased	Normal	Normal	[4]
Grade II	Variable	Moderately Increased	Slightly Decreased	Slightly Reduced	[18]
Grade III	Decreased	Highly Elevated	Significantly Decreased	Low	[16]

### Grade I (well-differentiated)

Tumors exhibit minimal iron dysregulation, maintaining relatively stable iron metabolism. In these tumors, serum iron, ferritin, transferrin saturation, and to TIBC levels typically remain within normal ranges. The limited alterations in iron homeostasis suggest a reduced dependency on iron for tumor proliferation, leading to a better prognosis and lower metastatic potential. These well-differentiated tumors tend to have a slower growth rate and respond favorably to conventional treatment modalities [4].

### Grade II (moderately differentiated)

Tumors demonstrate intermediate changes in iron metabolism, reflecting a moderate increase in cellular proliferation and metabolic activity. Serum ferritin levels may be slightly elevated, indicating increased iron sequestration within tumor cells to support their rapid growth. Transferrin saturation may show mild alterations, reflecting an enhanced iron uptake mechanism in these tumor cells. These changes contribute to a more aggressive phenotype compared to well-differentiated tumors, necessitating close monitoring for disease progression [18].

### Grade III (poorly differentiated)

Tumors exhibit significant iron dysregulation, contributing to an aggressive and metastatic phenotype. These tumors display markedly elevated ferritin levels, reflecting enhanced intracellular iron storage to sustain rapid tumor proliferation. Additionally, decreased transferrin saturation and increased oxidative stress from iron-mediated ROS production promote genomic instability and tumor progression [19]. Poorly differentiated tumors have a higher mitotic index and nuclear pleomorphism, correlating with increased metastatic potential and poorer clinical outcomes. The iron-enriched tumor microenvironment in these aggressive tumors further exacerbates tumor invasion and resistance to therapy [20].

## Association of serum iron panel with molecular immunophenotypes

Breast carcinoma is classified into molecular subtypes based on receptor expression patterns, including estrogen receptor (ER), progesterone receptor (PR), and human epidermal growth factor receptor 2 (HER2). These molecular subtypes exhibit distinct biological behaviors, with variations in iron metabolism playing a crucial role in tumor progression and prognosis [21].

### Luminal A (ER+/PR+/HER2-)

Tumors generally display lower levels of iron dysregulation. Although ferritin levels may be moderately elevated, the overall impact of iron metabolism on tumor progression remains minimal. These tumors are less aggressive, exhibit lower proliferative indices, and have a favorable prognosis. The mild alterations in iron homeostasis suggest that iron metabolism is not a major driver of tumor growth in this subtype [6].

### Luminal B (ER+/PR+/HER2 + or High Ki-67)

Tumors demonstrate increased iron utilization due to their highly proliferative nature. Elevated ferritin levels are commonly observed, correlating with enhanced tumor growth and metabolic activity. The increased demand for iron in these tumors supports their rapid division and survival, making iron metabolism a potential factor in disease progression. Luminal B tumors tend to be more aggressive than Luminal A and may require intensive therapeutic strategies [22].

### HER2-enriched (ER-/PR-/HER2+)

Tumors exhibit significant alterations in iron metabolism. Higher serum iron and ferritin levels are frequently detected, contributing to oxidative stress and increased tumor aggressiveness. Iron overload in these tumors may promote ROS production, leading to DNA damage and enhanced tumor progression. The association between HER2 overexpression and iron dysregulation suggests a potential link between HER2-driven tumorigenesis and iron metabolism [23].

### TNBC (ER-/PR-/HER2-)

Associated with the highest degree of iron dysregulation among all subtypes. Elevated ferritin levels, altered iron transport proteins, and disrupted iron homeostasis correlate with the aggressive behavior and poor prognosis of TNBC. Increased intracellular iron storage and oxidative stress contribute to enhanced tumor proliferation, invasion, and therapy resistance [6]. Given the critical role of iron in TNBC progression, targeting iron metabolism could offer promising therapeutic strategies for managing this aggressive breast cancer subtype. Understanding the relationship between serum iron panel parameters and molecular subtypes of breast carcinoma may provide valuable prognostic insights and facilitate the development of targeted therapies based on iron homeostasis [24].

## Mechanisms linking iron panel to breast cancer prognosis

**Fig. 2:**
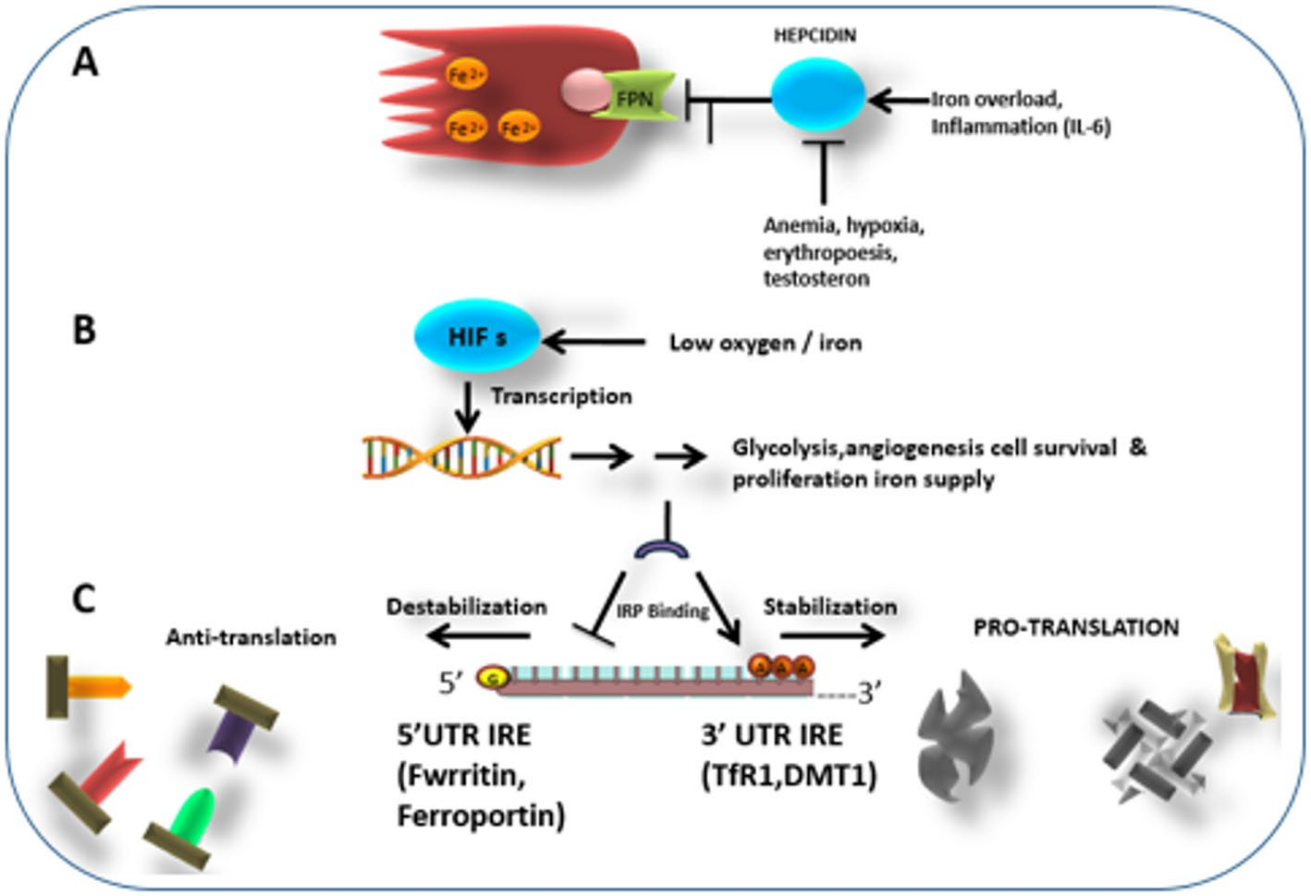
Molecular Mechanisms of Iron Dysregulation in Breast Cancer

Iron metabolism plays a critical role in breast cancer progression and prognosis, primarily through mechanisms involving oxidative stress, inflammation, and dysregulation of iron-regulatory proteins [24] see in Fig. 2.

### Iron overload and oxidative stress

Iron overload is a key contributor to breast cancer progression due to its role in generating ROS. These radicals induce oxidative stress, causing DNA damage, lipid peroxidation, and protein modifications, ultimately leading to genomic instability [10]. Additionally, oxidative stress creates a tumor-supportive microenvironment by modulating signaling pathways involved in cell survival and immune evasion. Cancer cells exhibit an increased demand for iron to sustain their rapid proliferation, further exacerbating iron overload and its deleterious effects. This self-perpetuating cycle contributes to the aggressive nature of certain breast cancer subtypes, particularly triple-negative breast cancer TNBC and HER2-enriched tumors, where iron dysregulation is most pronounced [25].

### Ferritin as an inflammatory marker

Ferritin, a key iron storage protein, serves as an important inflammatory marker in breast carcinoma. Elevated serum ferritin levels are frequently observed in cancer patients and are associated with increased tumor burden, systemic inflammation, and immune suppression [26]. Additionally, ferritin exerts immunosuppressive effects by modulating macrophage polarization, favoring tumor-associated macrophages (TAMs) that support tumor progression. Chronic inflammation, a hallmark of cancer, further drives iron accumulation by upregulating hepcidin, a key regulator of iron homeostasis. This leads to iron retention within tumor cells and a reduction in iron availability for normal physiological functions, thereby reinforcing tumor growth and metastatic potential [6].

### Iron-regulatory proteins and tumor growth

**Fig. 3:**
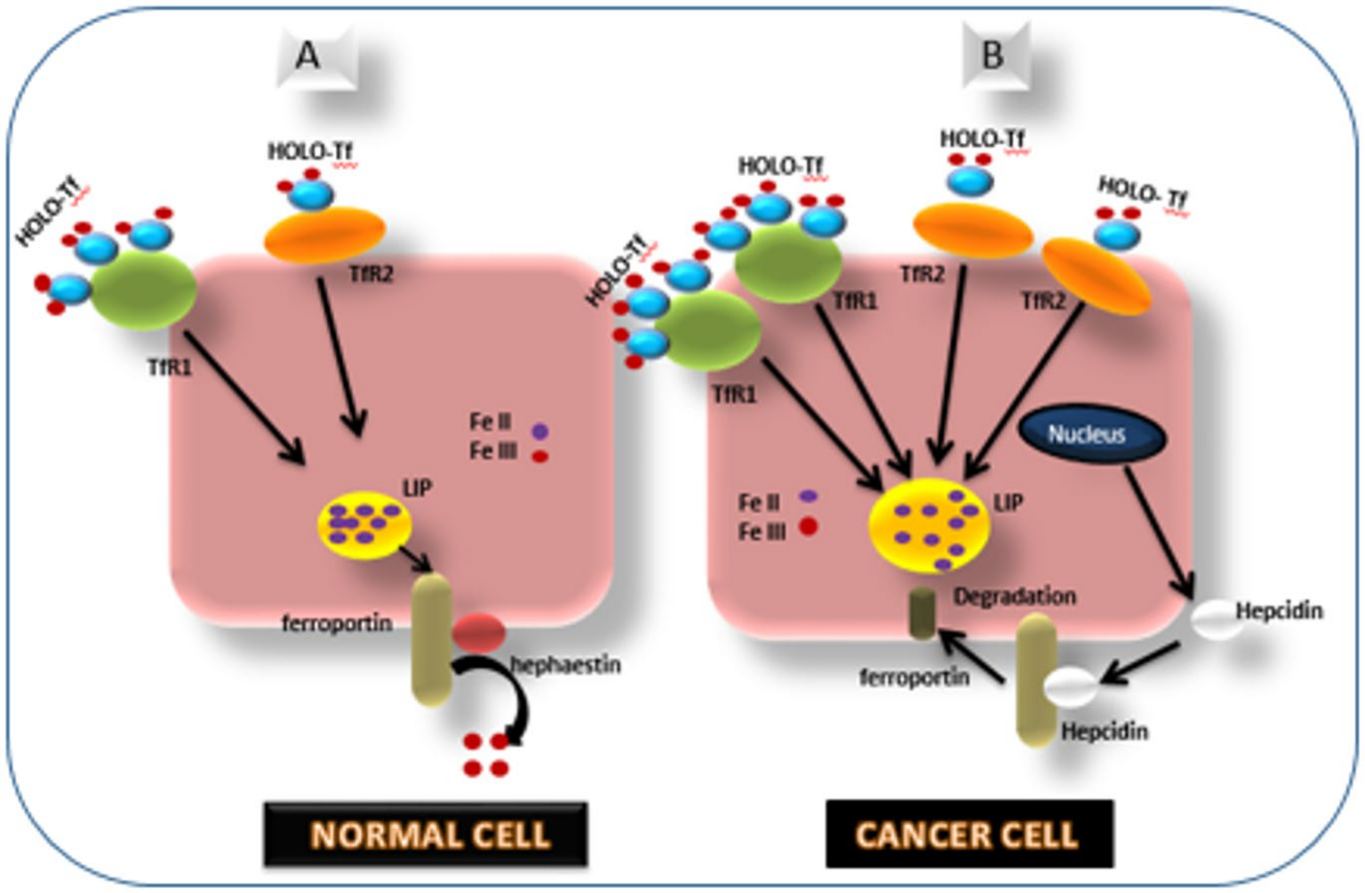
Iron-Regulatory Proteins and Tumor Growth

Iron homeostasis is maintained by a tightly regulated network of iron transport proteins is depicted in Fig. 3, including transferrin, TfR, ferroportin, and hepcidin. Dysregulation of these proteins has significant implications for breast cancer prognosis [27].

### TfR

Increased expression of transferrin receptor in tumor cells enhances iron uptake, fueling their metabolic demands and promoting aggressive behavior. Overexpression of TfR is associated with poor prognosis and resistance to therapy, making it a potential target for therapeutic intervention [28].

#### Ferroportin

Ferroportin is responsible for iron efflux from cells. In breast cancer, reduced ferroportin expression leads to intracellular iron accumulation, driving tumor progression. Low ferroportin levels correlate with increased tumor aggressiveness and poor patient outcomes [29].

#### Hepcidin

Hepcidin, a peptide hormone that regulates iron homeostasis, is frequently overexpressed in breast cancer. Elevated hepcidin levels inhibit ferroportin activity, trapping iron within tumor cells and contributing to tumor growth and metastasis [30].

The interplay between iron overload, ferritin levels, and iron-regulatory proteins influences tumor aggressiveness and patient outcomes. Targeting iron metabolism through therapeutic strategies such as iron chelation, ferroptosis-inducing drugs, and modulation of iron-regulatory proteins may offer novel approaches for breast cancer treatment [24].

## Clinical implications and future directions

### Prognostic biomarker potential

The serum iron panel, which includes parameters such as serum iron, ferritin, transferrin saturation, and total TIBC, holds promise as a non-invasive prognostic biomarker for breast cancer. Alterations in iron metabolism have been associated with tumor aggressiveness, oxidative stress, and immune evasion. Monitoring iron-related changes may assist in early detection, risk stratification, and prognosis prediction [4]. Elevated ferritin levels, in particular, correlate with increased tumor burden and poor clinical outcomes. Regular assessment of iron metabolism could complement existing histopathological and molecular markers, improving disease monitoring and treatment planning [19].

### Therapeutic targeting of iron metabolism

Given the strong link between iron dysregulation and cancer progression, targeting iron metabolism represents a novel therapeutic strategy see in Table 3. Iron chelators, such as deferoxamine, help reduce iron availability for tumor cells, potentially limiting their proliferation. Additionally, ferroptosis-inducing agents, which exploit iron-dependent cell death mechanisms, have emerged as promising anti-cancer therapies. Modulating key iron-regulatory proteins, such as hepcidin and ferroportin, may also offer new treatment avenues, particularly for aggressive subtypes like TNBC [31].

**Table 3:** Potential Therapeutic Strategies Targeting Iron Metabolism in Breast Cancer

Strategy	Mechanism	Clinical Implications	References
Iron Chelators	Reduces iron availability	Limits tumor growth	[32]
Ferroptosis Inducers	Enhances iron-dependent cell death	Targets therapy-resistant tumors	[33]
Hepcidin Modulators	Restores iron homeostasis	Improves patient outcomes	[34]

###  Personalized treatment strategies

Integrating iron metabolism data with genomic and proteomic analyses could enhance personalized treatment approaches for breast carcinoma. Identifying patient-specific iron metabolic signatures may help tailor therapies, optimize drug efficacy, and minimize resistance. Future research should focus on clinical trials investigating iron-targeted interventions and their role in combination with conventional therapies. A deeper understanding of iron metabolism in cancer could pave the way for more effective and individualized treatment strategies, improving patient outcomes in breast carcinoma [24].

## Future perspectives and research directions

Iron metabolism has emerged as a crucial factor influencing breast carcinoma progression, prognosis, and therapeutic response. However, further research is needed to establish the clinical utility of serum iron biomarkers and to explore novel iron-targeted interventions. Advancing our understanding of iron homeostasis in breast cancer will require interdisciplinary approaches integrating molecular, biochemical, and clinical research [6].

### Larger cohort studies for biomarker validation

To establish the prognostic significance of serum iron biomarkers in breast carcinoma, larger cohort studies across diverse populations are necessary. Current evidence suggests that iron parameters, such as ferritin, serum iron, transferrin saturation, and TIBC, correlate with tumor aggressiveness and patient outcomes. However, most studies are limited by small sample sizes and inconsistent methodologies. Large-scale, multicenter clinical trials are essential to validate these findings and standardize serum iron panel assessments for clinical use. Additionally, longitudinal studies tracking iron metabolism alterations across different stages of breast cancer could provide valuable prognostic insights [35].

### Mechanistic insights into iron-related gene expression

A deeper exploration of iron-related gene expression in breast carcinoma is crucial for understanding how iron metabolism influences tumor biology. Genes regulating iron uptake, storage, and export—such as TfR, ferroportin, hepcidin, and ferritin—play a pivotal role in tumor proliferation and metastasis. Investigating the epigenetic and transcriptional regulation of these genes may reveal novel therapeutic targets. Moreover, studying the role of iron-dependent pathways, such as ferroptosis, in breast cancer progression could open new avenues for treatment strategies aimed at selectively inducing cancer cell death [36].

### Integration with multi-omics approaches

A comprehensive understanding of iron metabolism in breast carcinoma can be achieved by integrating iron-related data with multi-omics approaches, including genomics, proteomics, metabolomics, and transcriptomics. Combining these datasets could provide a holistic view of how iron dysregulation interacts with other cancer hallmarks, such as inflammation, oxidative stress, and immune evasion. Artificial intelligence and machine learning models could further aid in identifying predictive iron-related biomarkers and therapeutic targets [37].

Future research should focus on large-scale biomarker validation, mechanistic insights into iron metabolism, and integration with multi-omics approaches to improve breast cancer prognosis and therapy. By bridging the gap between basic research and clinical applications, novel iron-targeted strategies could significantly enhance personalized treatment and patient outcomes in breast carcinoma [37].

## Limitations of the study

This review has several limitations that should be considered while interpreting the findings:

### Heterogeneity of included studies

The studies included in the review differed based on design, sample size, patient characteristics, and methodology, which may influence the consistency of reported results.

### Predominance of observational data

Most available evidence is based on observational and retrospective studies, limiting the ability to draw causal relationships between iron metabolism and breast carcinoma progression.

### Lack of standardized cut-off values

Variability in reference margins and laboratory evaluations for serum iron parameters diminishes comparability between studies.

### Limited subtype-specific data

Although some studies indicated that there are differences between molecular subtypes, exhaustive analyses of the subtypes, at the molecular level, are rarely adequate.

### Potential confounding factors

Iron metabolism may be affected by inflammation, nutritional status, liver function, and comorbidities whose factors are not appropriately accounted for in individual studies.

### Publication bias

Studies showing strong associations might be more likely to be published, potentially overestimating the strength of observed relationships.

## Conclusion

Based on existing evidence, changes in serum iron parameters including ferritin may underlie tumor aggressiveness and molecular subtypes of breast cancer. These findings suggest that iron metabolism may be involved in tumor biology. But this evidence is mostly based on heterogeneous and observational research studies, which means the generalizability of these associations is questionable. As such, serum iron biomarkers should be considered as potential adjunctive indicators rather than definitive prognostic tools at present. Further well-designed large-scale prospective studies in that area are needed for routine use in oncological practices in order to confirm their clinical utility.

## Data Availability

No datasets were generated or analysed during the current study.
